# P17 Orthopaedic arthroplasty antibiotic prophylaxis: a change in practice

**DOI:** 10.1093/jacamr/dlaf230.024

**Published:** 2025-12-04

**Authors:** I Galloway, K Hogan, R Walker

**Affiliations:** Royal Cornwall Hospital Trust, Truro, UK; Royal Cornwall Hospital Trust, Truro, UK; Royal Cornwall Hospital Trust, Truro, UK

## Abstract

**Background:**

Prosthetic Joint Infections (PJIs) are serious complications of orthopaedic surgery and result in a huge economic burden on the NHS. Perioperative antibiotic prophylaxis is a safe, effective and low-cost intervention that aims to reduce the risk of PJIs. There is widespread variation across UK NHS trusts regarding which antibiotic prophylaxis is used with gentamicin and teicoplanin (G&T) being most commonly used. An increase in number of PJIs had been noted in our NHS trust, and so our prophylaxis protocol was reviewed. It was suggested that G&T would be a more appropriate broad-spectrum cover than the current flucloxacillin and gentamicin (F&G) regime.

**Objectives:**

A retrospective review was conducted to identify all recent PJIs treated, common organisms and sensitivities, and whether a change in antibiotic protocol could have prevented this complication.

**Methods:**

A retrospective review over a 12 month period identified all patients treated for PJIs within six months of their primary procedure. Patient details were collected from orthopaedic-microbiology multi-disciplinary teams (MDTs) and operating lists. Patient records were used to gather details of their initial procedure, including peri-operative antibiotic prophylaxis, causative organisms isolated, antibiotic resistance and definitive treatment.

**Results:**

14 patients were identified as being treated for PJIs during the period, aged 58–91 years (median 80). 57% (8/14) were elective admissions. Procedures included total hip replacements (5/14), hip hemiarthroplasty (3/14), total knee replacements (3/14) and open reduction internal fixations for periprosthetic fractures (3/14). 71% (10/14) were primary surgeries, whilst two revisions were for trauma, one revision for aseptic loosening and one was a second stage revision after previously cleared infection. 93% (13/14) received F&G as perioperative prophylaxis, as per trust guidelines. One patient received G&T due to a penicillin allergy. All PJIs were treated with antibiotic therapy, with 86% patients (12/14) having further surgery. These included debridement with implant retention (36%), debridement with implant exchange (36%), or debridement with removal of the implant entirely (14%). All patients had samples taken to identify the causative organisms. 19 positive samples identified 14 different bacteria, with two samples not isolating any organisms despite clear clinical signs of PJI. 43% (6/14) of patients had polymicrobial infections. The most common organism isolated was *Staphylococcus aureus* (29%), followed by *Staphylococcus epidermidis* and *Group G Streptococcus* (both 14%). The local microbiology team reviewed all positive samples and determined that 76% (11/14) of infections were sensitive to Teicoplanin, whilst only 50% (7/14) were sensitive to Flucloxacillin.

**Conclusions:**

Based on this finding, the Orthopaedic team were happy to support a change in perioperative prophylaxis from F&G to G&T, in the hope of reducing local PJI rates. The impact of this change will be monitored on an annual basis to help guide further policy updates.

